# Determination of reliable lung function parameters in intubated mice

**DOI:** 10.1186/s12931-019-1177-9

**Published:** 2019-09-14

**Authors:** Eline Bonnardel, Renaud Prevel, Marilyne Campagnac, Marielle Dubreuil, Roger Marthan, Patrick Berger, Isabelle Dupin

**Affiliations:** 1 Univ-Bordeaux, Centre de Recherche Cardio-thoracique de Bordeaux, U1045, Département de Pharmacologie, CIC 1401, F-33000 Bordeaux, France; 2 INSERM, Centre de Recherche Cardio-thoracique de Bordeaux, U1045, CIC 1401, F-33000 Bordeaux, France; 3 CHU de Bordeaux, Service d’exploration fonctionnelle respiratoire, CIC 1401, F-33604 Pessac, France

**Keywords:** Lung function, Mice model, Asthma, COPD, Orotracheal intubation

## Abstract

**Background:**

Animal models and, in particular, mice models, are important tools to investigate the pathogenesis of respiratory diseases and to test potential new therapeutic drugs. Lung function measurement is a key step in such investigation. In mice, it is usually performed using forced oscillation technique (FOT), negative pressure-driven forced expiratory (NPFE) and pressure-volume (PV) curve maneuvers. However, these techniques require a tracheostomy, which therefore only allows end-point measurements. Orotracheal intubation has been reported to be feasible and to give reproducible lung function measurements, but the agreement between intubation and tracheostomy generated-data remains to be tested.

**Methods:**

Using the Flexivent system, we measured lung function parameters (in particular, forced vital capacity (FVC), forced expiratory volume in the first 0.1 s (FEV0.1), compliance (Crs) of the respiratory system, compliance (C) measured using PV loop and an estimate of inspiratory capacity (A)) in healthy intubated BALB/cJ mice and C57BL/6 J mice and compared the results with similar measurements performed in the same mice subsequently tracheostomized after intubation, by means of paired comparison method, correlation and Bland-Altman analysis. The feasibility of repetitive lung function measurements by intubation was also tested.

**Results:**

We identified parameters that are accurately evaluated in intubated animals (i.e., FVC, FEV0.1, Crs, C and A in BALB/cJ and FVC, FEV0.1, and A in C57BL/6 J). Repetitive lung function measurements were obtained in C57BL/6 J mice.

**Conclusion:**

This subset of lung function parameters in orotracheally intubated mice is reliable, thereby allowing relevant longitudinal studies.

**Supplementary information:**

**Supplementary information** accompanies this paper at (10.1186/s12931-019-1177-9).

## Background

Chronic airway diseases e.g. chronic obstructive pulmonary disease (COPD) are devastating diseases characterized by impaired respiratory function. For instance, parameters derived from the forced expiratory maneuvers, such as the forced expiratory volume in the first second (FEV1) to the forced vital capacity (FVC) ratio are widely used to characterize functional obstruction [1]. COPD can also be complicated by the destruction of the parenchymal tissue (i.e., emphysema) or by a fibrotic tissue transformation (i.e., peribronchial fibrosis), which are evidenced by modifications of lung compliance. Small animal models, that recapitulate the hallmark features of the disease, are of major interest to gain insights into chronic respiratory diseases development and to test potential therapeutic drugs. Although other animals such as dogs, guinea pigs or rats have also been used as models of chronic respiratory disease [2–4], mice models offer unique opportunities related to deep knowledge of mouse biology, and possibility of genetic manipulation [5].

Similar to spirometry in cooperative humans, equipment for lung function testing such as the Buxco or the Flexivent system has been developed for mice. Inflating mouse lungs to a given pressure followed by connection of animal’s airways to a negative pressure reservoir allows to trigger a forced expiratory maneuver and thus to measure FEV0.1 (forced expiratory volume in the first 0.1 s), FVC (forced vital capacity), and the surrogate of the human FEV1/FVC ratio, so-called FEV0.1/FVC ratio [6]. Single frequency Forced Oscillation Technique (FOT) measurements fitted to single compartment model allow to only measure resistance (Rrs) and compliance (Crs) of the respiratory system, whereas broadband FOT measurements fitted to constant phase model distinguish airway and tissue mechanics by quantifying tissue damping (G), tissue elastance (H) and the resistance of the central airways (Rn) [7]. Finally, lung distensibility (compliance, C, also called in some other studies “quasi-static compliance”, Cst) can be characterized by inflating and deflating the lungs with discrete steps to draw a partial pressure-volume (PV) loop [8].

However, monitoring lung function in mice, as above described, requires a tracheostomy, a procedure that has hampered longitudinal studies in animal models of chronic respiratory diseases. Orotracheal intubation could overcome this problem although the small size of the mouse, presenting numerous advantages such as cheap housing and cost minimizing for testing expensive drugs, is challenging for this technique [7]. However, numerous studies have proposed methods to intubate mice, starting from Ho et al. [9] to more recent ones [10–15]. Since the use of a cannula compatible with lung function measurement further complicates the process, few studies report lung function measurement in intubated mice. Respiratory resistance and compliance have been measured using orotracheal intubation [16]. Repetitive measurements of lung resistance and dynamic compliance have been obtained in orotracheally intubated healthy and allergic BALB/c mice [17, 18]. The study of Das et al. reported repetitive monitoring of respiratory resistance in intubated C57BL/6 J mice [19]. Orotracheal intubation provides reproducible lung function measurements in healthy BALB/cJ mice using the Buxco system [20]. However, on the one hand, the agreement between intubation and tracheostomy generated-data has not been properly tested so far and, on the other hand, mice strains exhibit different lung mechanical properties [21]. Therefore, evaluating lung function using intubation in comparison with the standard method still remains to be tested in different mice strains [22].

In two inbred mice strains that are routinely studied (i.e., the BALB/cJ and C57BL/6 J mice), we thus aimed at comparing lung function measurements obtained using orotracheal intubation with the same data obtained using tracheostomy. We evaluated the agreement between the two different methods by means of intra-class correlation and Bland-Altman analyses. We also tested the feasibility of repetitive lung function measurements by intubation.

## Methods

### Animals

Male BALB/cJ mice (~ 25 g, 10–11 weeks old) and male C57BL/6 J mice (~ 25 g, 10–11 weeks old) were obtained from Janvier (St Berthevin, France). The mice were housed in a conventional animal facility with free access to food and water. All animal studies were performed according to European and French directives about vertebrate animals protection use for animal experiments. Agreement was obtained from French authorities (number A33–063-907) and all the protocols used were approved by the local ethics committee (“Comité d’éthique regional d’Aquitaine”, protocol number: 2018030715546195–APAFiS # 13957).

### Lung function measurement

Mice were anesthetized with an intraperitoneal injection of 125 mg/kg ketamine and 10 mg/kg xylazine (Centravet, Dinan, France). The same anesthetic protocol was used for intubation and tracheostomy. Orotracheal intubation was performed using an 18G, 30 mm intravenous plastic catheter previously cut and tapered at 20 mm (BD Insyte, San Jose, CA), without suture sealing the wall of the trachea around the intubation catheter. Tracheostomy was subsequently performed using either the 18G metal cannula or the intubation catheter, with a suture sealing the wall of the trachea around the cannula. The animal was then connected to the small animal ventilator (flexiVent, Scirecq) and mechanically ventilated at a respiratory rate of 150 breaths/min, a tidal volume of 10 mL/kg and a PEEP set at 3 cmH_2_O. Forced oscillation measurements were performed using the single-FOT maneuver (“Snapshot-150 perturbation”) and the broadband FOT maneuver (“Quick Prime-3 perturbation”). The single FOT measurements were fitted to single compartment model to determine respiratory system resistance (Rrs) and respiratory system compliance (Crs). The multi-frequency FOT measurements were fitted to constant phase model to obtain newtonian resistance (Rn), tissue damping (G) and tissue elastance (H). PV loops were also generated to obtain compliance (C) of the respiratory system, an estimate of inspiratory capacity (A), curvature of the upper portion of the deflation limb of the PV curve (K) and the area enclosed by the PV loop (Area). The negative pressure-driven forced expiratory (NPFE) maneuver was then performed by inflating the mouse lungs to a pressure of 30 cm H_2_O over 1 s, hold this pressure for 2 s before connecting the animal’s airways to the negative pressure reservoir (− 50 cm H_2_O) for 2 s. The forced expired volume over 0.1 s (FEV0.1), forced vital capacity (FVC) and the peak expiratory flow (PEF) were calculated directly from the flow-volume loop generated during lung deflation. In every mouse, each maneuver was repeated until 2 acceptable measurements (coefficient of determination ≥0.95) were recorded. The median of at least 2 acceptable measurements was then calculated.

### Experimental design for validation of intubation

The order of the experiment was randomized for C57BL/6 J and BALB/cJ mice. The Flexivent was re-calibrated for the different cannulas used in intubated and tracheostomized mice if necessary. Lung function measurements were performed first in intubated mice and immediately after in the same tracheotomized animal, either using different cannulas or using the same cannula (i.e., intubation catheter). The measurements performed in tracheostomized mouse were compared with those previously recorded in the same intubated mouse. To test the impact of previous intubation on lung function measured on tracheotomized C57BL/6 J mice, other lung function measurements were performed on either solely intubated mice or solely tracheotomized animals. The measurements performed in tracheostomized mice were compared with those obtained in intubated mice. Mice were then sacrificed through pentobarbital injection of 500 mg/kg.

### Experimental design for repeated intubations

The study protocol is shown Fig. 9a. C57BL/6 J mice were anesthetized and intubated as described above at day 0. After lung function measurement, the mice were extubated. Mice were weighed every 3–4 days and general health was checked daily. Nineteen days after the first intubation, lung function is measured using a second intubation. Mice were then sacrificed through pentobarbital injection of 500 mg/kg.

### Statistical analysis

The statistical analysis was performed with Prism 6 software (GraphPad, La Jolla, CA). Statistical significance, defined as *P* < 0.05, was analyzed using paired or unpaired t tests for variables with parametric distribution, or using Wilcoxon or Mann–Whitney tests for variables with nonparametric distribution. The linear relationship between measurements obtained in intubated and tracheostomized mice was evaluated by Pearson correlation. Bland-Altman plots were built with Prism 6 software. The relationship between the difference and the mean of the two methods (orotracheal intubation and tracheostomy) was evaluated by Spearman correlation.

## Results

Because the metal cannula used for tracheostomy was traumatic when used for orotracheal intubation, we tested other intubation catheters, and finally chose the cannula that offered the best compromise between a resistance close to that of the tracheostomy cannula and sufficient flexibility to enable orotracheal intubation. To facilitate intubation, the 18G catheter was narrowed by slightly heating the distal end of the tube. The resistance of the tube, measured during calibration of the FlexiVent system was 0.32 cmH_2_O.s/mL. This value was close to that of 18G metal cannula used for tracheostomy (0.27 cmH_2_O.s/mL). One of the 15 BALB/cJ mice (7%) and 5 of the 15 C57BL/6 J mice (33%) died during the intubation procedure. One of the 15 BALB/cJ mice (7%) was successfully intubated, but showed aberrant lung function data using intubation as well as tracheostomy. On average, 23 min and 26 min separated the measurements performed following intubation from those following tracheostomy in C57BL/6 J and BALB/cJ mice, respectively. No sign of respiratory drive was observed during the measurements. Overall, lung function was successfully measured in 13 of the 15 BALB/cJ mice (87%) and 10 of the 15 of the C57BL/6 J mice (67%) in response to NPFE maneuver, in 14 of the 15 BALB/cJ mice (93%) and 10 of the 15 of the C57BL/6 J mice (67%) using FOT single compartment model and PV loop maneuver, and in 12 of the 15 BALB/cJ mice (80%) and 9 of the 15 of the C57BL/6 J mice (60%) using FOT constant phase model. We then compared the lung function data obtained using intubation and subsequently tracheostomy in the same mice of each strains.

**Fig. 1:**
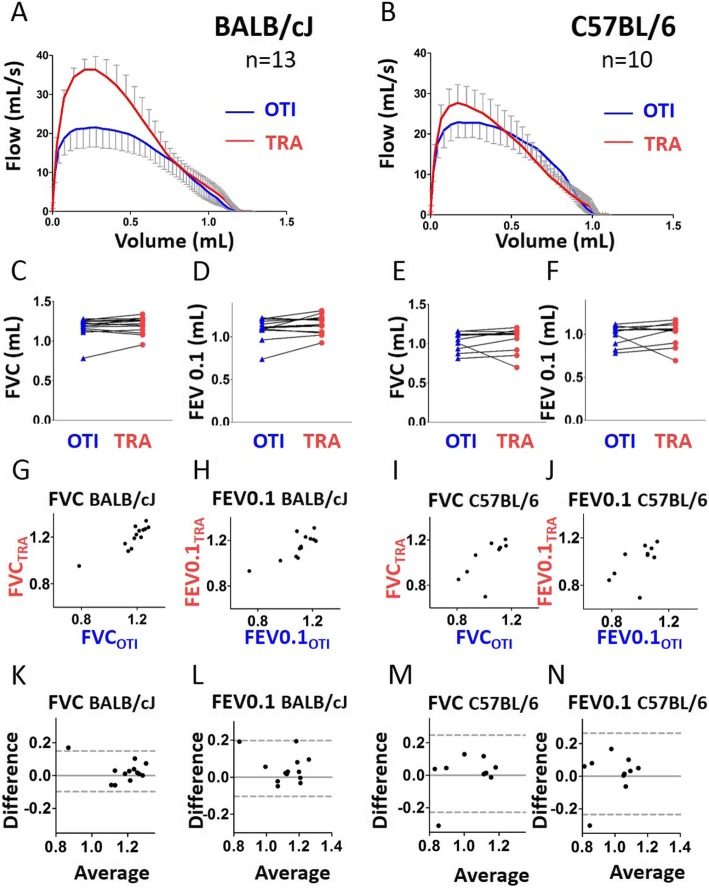
Evaluation of lung function measurement assessed by the NPFE maneuver in mice using orotracheal intubation and tracheostomy. **a**, **b**, Average expiratory flow-volume curves of intubated (“OTI”, in blue) and tracheostomized (“TRA”, in red) mice. n = 13 BALB/cJ mice, n = 10 C57BL/6 J mice. Lower and upper error bars represent standard deviations for intubation and tracheostomy, respectively. **c**-**f**, Forced vital capacity (“FVC”, **c** and **e**), forced expired volume over 0.1 s (“FEV0.1”, **d** and **f**) in intubated and tracheostomized BALB/cJ mice (**c**, **e**) and C57BL/6 J mice (**d**, **f**). Data represent individual mice and are analyzed by the Wilcoxon signed-rank test or paired t tests. * *P* < 0.05. **g**, **i**, Relationships between FVC measured in intubated and tracheostomized BALB/cJ (**g**) and C57BL/6 J (**i**) mice. **h**, **j**, Relationships between FEV0.1 measured in intubated and tracheostomized BALB/cJ (**h**) and C57BL/6 J (**j**) mice. **k**-**n**, Bland-Altman plots to compare two measurements techniques for FVC in BALB/cJ (**k**) and C57BL/6 J (**m**), FEV0.1 in BALB/cJ (**l**) and C57BL/6 J (**n**). The upper and lower limits of agreement (95% confidence interval) are shown by a gray dotted line

Regarding BALB/cJ mice, the expiratory flow-volume curve was downward shifted especially at the onset of expiration in intubated compared with tracheostomized mice (Fig. 1a). As a consequence, the peak expiratory flow (PEF) and the FEV0.1/FVC ratio were significantly decreased in intubated compared to tracheostomized BALB/cJ mice (Additional file 1: Figure S1A-B). Of note, the magnitude of FEV0.1/FVC ratio decrease was very small (around 2%), and does not probably reflect a physiologically significant change. Regarding C57BL/6 J mice, by contrast, the flow-volume curve obtained in intubated mice was similar to that measured in tracheostomized animals (Fig. 1b). As a consequence, we did not find any significant difference between intubated and tracheostomized C57BL/6 J mice for PEF and FEV0.1/FVC (Additional file 1: Figure S1A-B). For both strains, FVC and FEV0.1 were not different between intubated and tracheostomized animals (Fig. 1c-f). FVC of C57BL/6 J and BALB/cJ mice, and FEV0.1 of BALB/cJ mice measured under the 2 conditions were also positively and significantly correlated (Fig. 1g-j and Additional file 1: Table S1). The difference between the two measurements (“bias”), calculated by Bland-Altman analysis was close to 0 (Additional file 1: Table S2), even if, for FVC in BALB/cJ mice, one point is outside the confidence interval (Fig. 1k-n). Moreover, there was no correlation between the difference and the average in the two conditions for FVC and FEV0.1 (Additional file 1: Table S3). Thus, the measurement of both FVC and FEV0.1 using orotracheal intubation can be considered accurate.

To evaluate the possibility that lung function measured in tracheotomized C57BL/6 J mice could have been affected by previous intubation, we compared lung function obtained in animals that have been only intubated or tracheostomized. We observed a downward shift of the expiratory flow-volume curve for intubated C57BL/6 J animals compared with tracheostomized mice (Additional file 1: Figure S2A), with a subsequent decrease in peak expiratory flow (PEF), FEV0.1, FVC and FEV0.1/FVC ratio in intubated C57BL/6 J mice compared to tracheostomized mice (Additional file 1: Figure S2B-E).

**Fig. 2:**
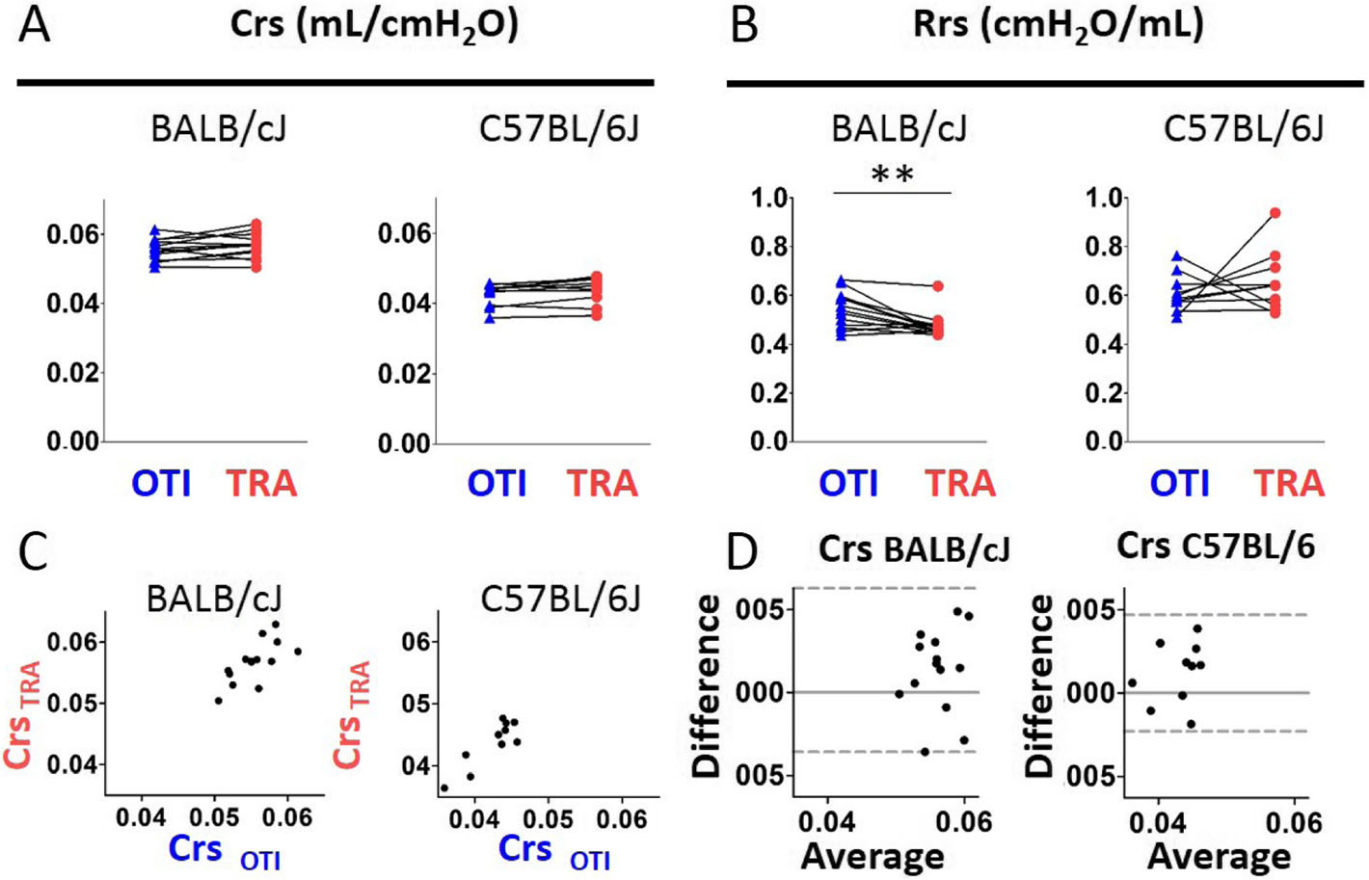
Evaluation of lung function measurement assessed by single compartment model obtained in mice using orotracheal intubation and tracheostomy. **a**-**b**, Comparaison of the variables compliance (“Crs”, **a**) and resistance (“Rrs”, **b**) of the respiratory system in intubated (“OTI”) and tracheostomized (“TRA”) mice. n = 14 BALB/cJ mice, n = 10 C57BL/6 J mice. Data represent individual mice and are analyzed by the Wilcoxon signed-rank test or paired t tests. ** *P* < 0.01. **c**, Relationships between Crs measured in intubated and tracheostomized BALB/cJ and C57BL/6 J mice. **d**, Bland-Altman plots to compare two measurements techniques for Crs. The upper and lower limits of agreement (95% confidence interval) are shown by a gray dotted line

Using single frequency FOT measurements fitted to the single compartment model, we found in both strains of mice a significant decrease of the coefficients of determination (COD) in intubated mice compared to tracheostomized animals (Additional file 1: Figure S3A). Excepted respiratory system resistance (Rrs) which was increased in intubated BALB/cJ mice, neither Rrs in C57BL/6 J mice nor respiratory system compliance (Crs) in both strains of mice, was different between intubated and tracheostomized animals (Fig. 2a-b). The presence of a significant positive association (Additional file 1: Table S1) together with the result of Bland-Altman analysis (Fig. 2c-d and Additional file 1: Table S2) and the absence of correlation between the difference and the average (Additional file 1: Table S3) indicated that the measurement of Crs using orotracheal intubation was accurate.

**Fig. 3:**
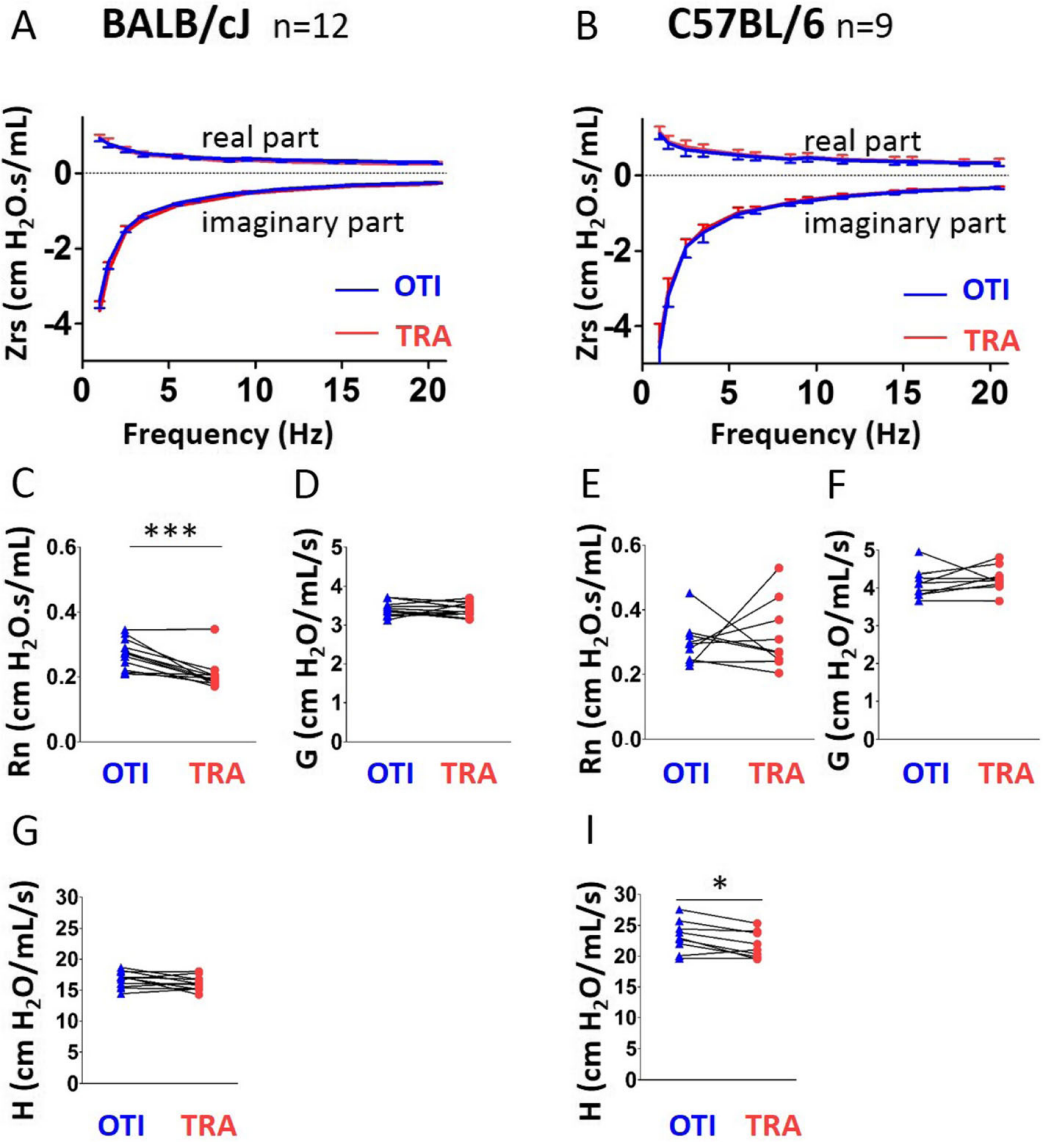
Evaluation of lung function measurement assessed by constant phase model obtained in mice using orotracheal intubation and tracheostomy. **a**, **b**, Average real (top) and imaginary (bottom) parts of respiratory system input impedance (Zrs) obtained in intubated (“OTI”, in blue) mice and tracheostomized (“TRA”, in red) mice. n = 12 BALB/cJ mice (**a**), n = 9 C57BL/6 J mice (**b**). Lower and upper error bars represent standard deviations respectively for respectively for intubation and tracheostomyintubation and tracheostomy. **c-i**, newtonian resistance (“Rn”, **c** and **e**), tissue damping (“G”, **d**, **f**), tissue elastance (“H”, **g**, **i**) in intubated and tracheostomized BALB/cJ mice (**c**, **d**, **g**) and C57BL/6 J mice (**e**, **f**, **i**). Data represent individual mice and are analyzed by the Wilcoxon signed-rank test or paired t tests. * *P* < 0.05, *** *P* < 0.001

At first glance, the impedance curves obtained by multi-frequency FOT in intubated BALB/cJ and C57BL/6 J mice appeared almost similar to those obtained with tracheostomy (Fig. 3a-b). However, they revealed some subtle differences: in BALB/cJ mice, the real part of the recorded in tracheostomized BALB/cJ mice was slightly above the same curve obtained intubated BALB/cJ mice at low frequency (1 Hz), and slightly below at higher frequencies (1.5–20.5 Hz). In contrast, in C57BL/6 J mice, the real part of the curve obtained in tracheostomized mice was always slightly above the same the curve obtained in intubated mice. Finally, calculation of the threshold frequency that discriminates the central compartment alone (at high frequency) from the combination central and distal compartments (at low frequency) showed that it was different in both strains. In this connection, the newtonian resistance (Rn, Fig. 3c) derived from the real part was increased in intubated BALB/cJ mice as was tissue elastance ( Fig. 3h, i) in intubated C57BL/6 J mice, compared corresponding parameters in tracheostomized animals. With the exception of H in C57BL/6 J mice, there was no significant correlation between Rn, G, H assessed under the two conditions (Additional file 1: Table S1). Although the average number of measurements required to get 2 coefficients of determination for the constant phase model fit (CODcp) values above 0.95 in intubated mice (*i.e*, 4.6 for BALBc/J and 5.3 for C57BL/6) was higher than that required in tracheotomized mice (*i.e*, 3.2 for BALBc/J and 3.6 for C57BL/6), this only represented a significant change in BALBc/J mice. The analysis of CODcp did not show any difference in intubated mice compared to tracheostomized animals (Additional file 1: Figure S3B).

**Fig. 4:**
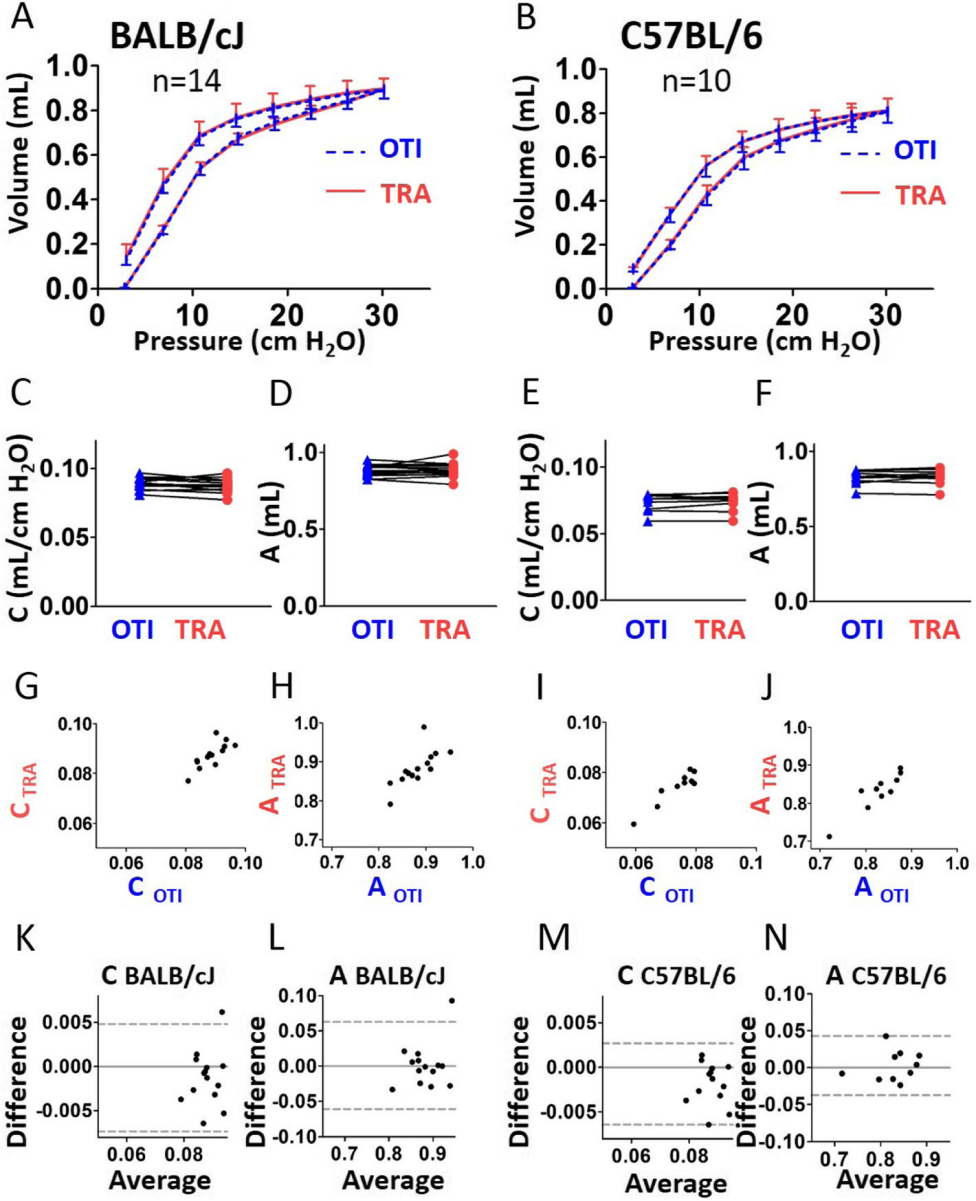
Evaluation of lung function measurement assessed by the Pressure-Volume (PV) curve maneuver in mice using orotracheal intubation and tracheostomy. **a**, **b**, Average PV curves of intubated (“OTI”, in blue) mice and tracheostomized (“TRA”, in red) mice. n = 14 BALB/cJ mice (**a**), n = 10 C57BL/6 J mice (**b**). Lower and upper error bars represent standard deviations for intubation and tracheostomy, respectively. **c**-**f**, compliance (“C”, **c** and **e**), estimate of inspiratory capacity (“A”, **d** and **f**) in intubated and tracheostomized BALB/cJ mice (**c**, **e**) and C57BL/6 J mice (**d**, **f**). Data represent individual mice and are analyzed by the Wilcoxon signed-rank test or paired t tests. **g**, **i**, Relationships between **c** measured in intubated and tracheostomized BALB/cJ (**g**) and C57BL/6 J (**i**) mice. **h**, **j**, Relationships between A measured in intubated and tracheostomized BALB/cJ (**h**) and C57BL/6 J (**j**) mice. **k**-**n**, Bland-Altman plots to compare two measurements techniques for C in BALB/cJ (**k**) and C57BL/6 J (**m**), A in BALB/cJ (**l**) and C57BL/6 J (**n**). The upper and lower limits of agreement (95% confidence interval) are shown by a gray dotted line

The PV curves recorded in intubated BALB/cJ and C57BL/6 J mice were very similar to those obtained with tracheostomy (Fig. 4a-b). The quality of the Salazar-Knowles model fit was unchanged in intubated mice as shown by the very similar coefficients of determination for the Salazar-Knowles model (CODsk) in intubated and tracheostomized animals (Additional file 1: Figure S3C). The compliance (C, Fig. 4c, e), the estimate of inspiratory capacity (A, Fig. 4d, f), the curvature of the upper portion of the deflation limb of the PV curve (K, Additional file 1: Figure S4A) and the area enclosed by the pressure volume loop (Area, Additional file 1: Figure S4B) were not significantly altered in intubated compared to tracheotomized mice. The result of the combination of correlation and Bland-Altman analysis thus indicated that C and A could be accurately measured in intubated mice (Fig. 4g-n, and Additional file 1: Tables S1, S2 and S3). Of note, C did not behave differently from Crs from a qualitative point of view: although the difference between C and Crs was always positive (close to 0.03 mL/cm H_2_O), C was strongly positively correlated with Crs in both strains, whatever the measurement method, orotracheal intubation or tracheostomy (Additional file 1: Figure S5B).

To distinguish the effect due to the cannula from that due to the procedure (i.e., tracheostomy vs intubation), we also performed another set of experiments using the same cannula. Because the metal cannula used for tracheostomy was traumatic when used for orotracheal intubation, we chose the intubation catheter for both procedures. Two of the 22 BALB/cJ mice (9%) died during the intubation procedure; 3 of the 22 BALB/cJ mice (14%) and 5 of the 22 C57BL/6 J mice (23%) could not be intubated. One of the 22 BALB/cJ mice (4%) was successfully intubated, but showed aberrant lung function data using intubation as well as tracheostomy. Overall, lung function was successfully measured in 16 of the 22 BALB/cJ mice (73%) and 16 of the 22 of the C57BL/6 J mice (73%) in response to NPFE maneuver, in 16 of the 22 BALB/cJ mice (73%) and 17 of the 22 of the C57BL/6 J mice (77%) using FOT single compartment model and PV loop maneuver, and in 8 of the 22 BALB/cJ mice (36%) and 15 of the 22 of the C57BL/6 J mice (68%) using FOT constant phase model.

**Fig. 5:**
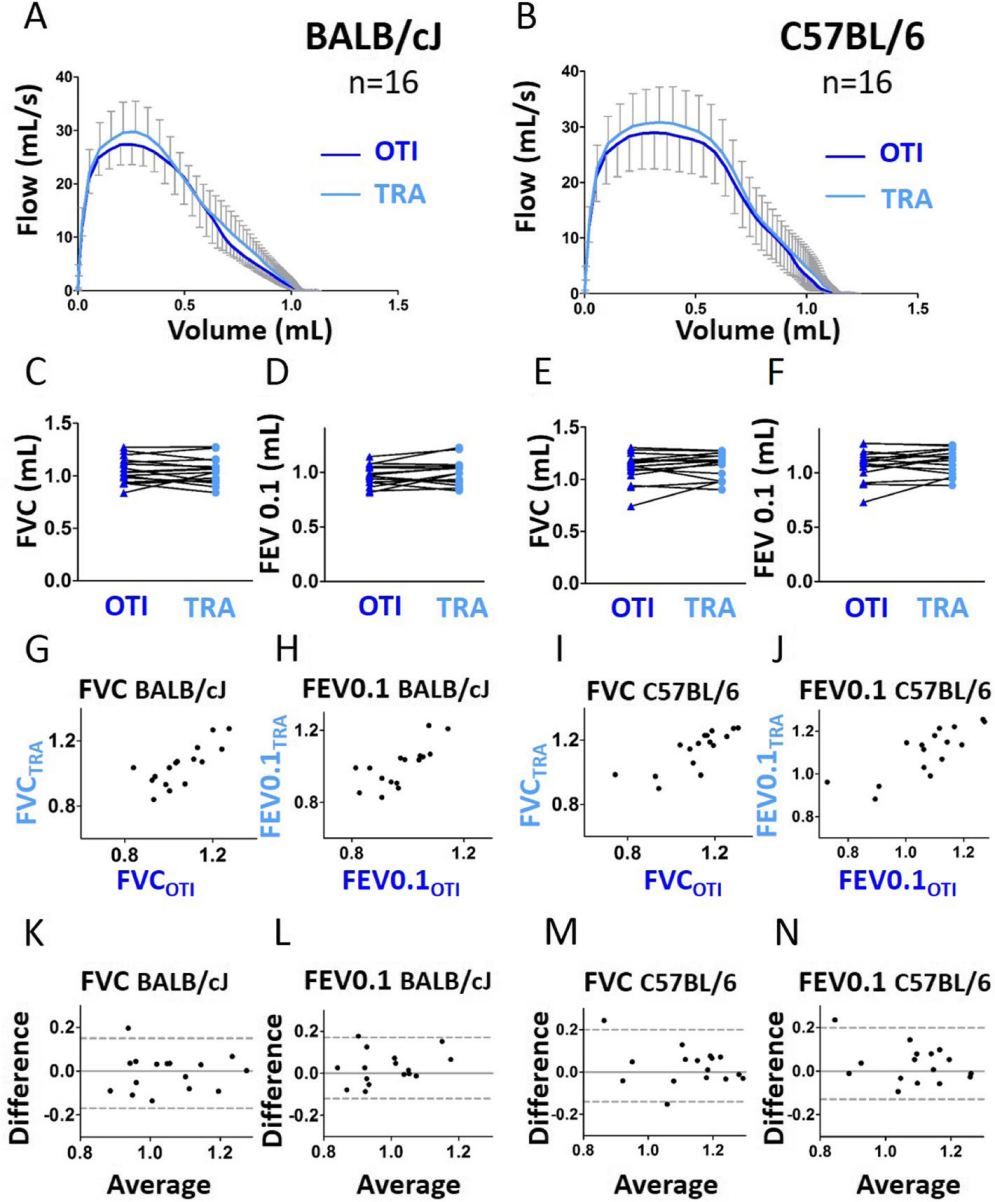
Evaluation of lung function measurement assessed by the NPFE maneuver in mice using orotracheal intubation and tracheostomy, both using the intubation cannula. **a**, **b**, Average expiratory flow-volume curves of intubated (“OTI”, in blue) and tracheostomized (“TRA”, in red) mice. n = 16 BALB/cJ mice, n = 16 C57BL/6 J mice. Lower and upper error bars represent standard deviations for intubation and tracheostomy, respectively. **c**-**f**, Forced vital capacity (“FVC”, **c** and **e**), forced expired volume over 0.1 s (“FEV0.1”, **d** and **f**) in intubated and tracheostomized BALB/cJ mice (**c**, **e**) and C57BL/6 J mice (**d**, **f**). Data represent individual mice and are analyzed by the Wilcoxon signed-rank test or paired t tests. **g**, **i**, Relationships between FVC measured in intubated and tracheostomized BALB/cJ (**g**) and C57BL/6 J (**i**) mice. **h**, **j**, Relationships between FEV0.1 measured in intubated and tracheostomized BALB/cJ (**h**) and C57BL/6 J (**j**) mice. **k**-**n**, Bland-Altman plots to compare two measurements techniques for FVC in BALB/cJ (**k**) and C57BL/6 J (**m**), FEV0.1 in BALB/cJ (**l**) and C57BL/6 J (**n**). The upper and lower limits of agreement (95% confidence interval) are shown by a gray dotted line

**Fig. 6:**
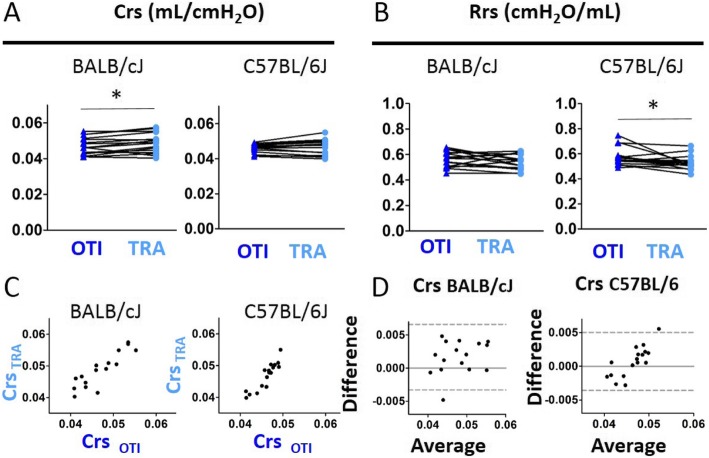
Evaluation of lung function measurement assessed by single compartment model obtained in mice using orotracheal intubation and tracheostomy, both using the intubation cannula. **a**-**b**, Comparaison of the variables compliance (“Crs”, **a**) and resistance (“Rrs”, **b**) of the respiratory system in intubated (“OTI”) and tracheostomized (“TRA”) mice. n = 16 BALB/cJ mice, n = 17 C57BL/6 J mice. Data represent individual mice and are analyzed by the Wilcoxon signed-rank test or paired t tests. * *P* < 0.05. C, Relationships between Crs measured in intubated and tracheostomized BALB/cJ and C57BL/6 J mice. **d**, Bland-Altman plots to compare two measurements techniques for Crs. The upper and lower limits of agreement (95% confidence interval) are shown by a gray dotted line

In both strains of mice, the flow-volume curves obtained in intubated mice were similar to those measured in tracheostomized animals (Fig. 5a-b). Except for the FEV0.1/FVC ratio which was slightly and significantly increased in tracheostomized BALB/cJ mice (Additional file 1: Figure S6B), FVC, FEV0.1 (Fig. 5c-f) and PEF (Additional file 1: Figure S6A) were not different between intubated and tracheostomized animals. Agreement analyses confirmed that the measurements of FVC and FEV0.1 using orotracheal intubation were accurate (Fig. 5g-n and Additional file 1: Tables S4, S5 and S6). Using single frequency FOT measurements fitted to the single compartment model, we could also confirm our previous results obtained with different cannulas, i.e., the validation of Crs but not Rrs assessment in intubated BALB/cJ mice (Fig. 6 and Additional file 1: Tables S4, S5 and S6). By contrast, the existence of a positive significant correlation between the average and the difference of both Crs and C measurements using intubation and tracheostomy in C57BL/6 J mice suggests that these sole parameters should not be evaluated using intubation in this mouse strain (Fig. 6d, Fig. 8m and Additional file 1: Table S6).

**Fig. 7:**
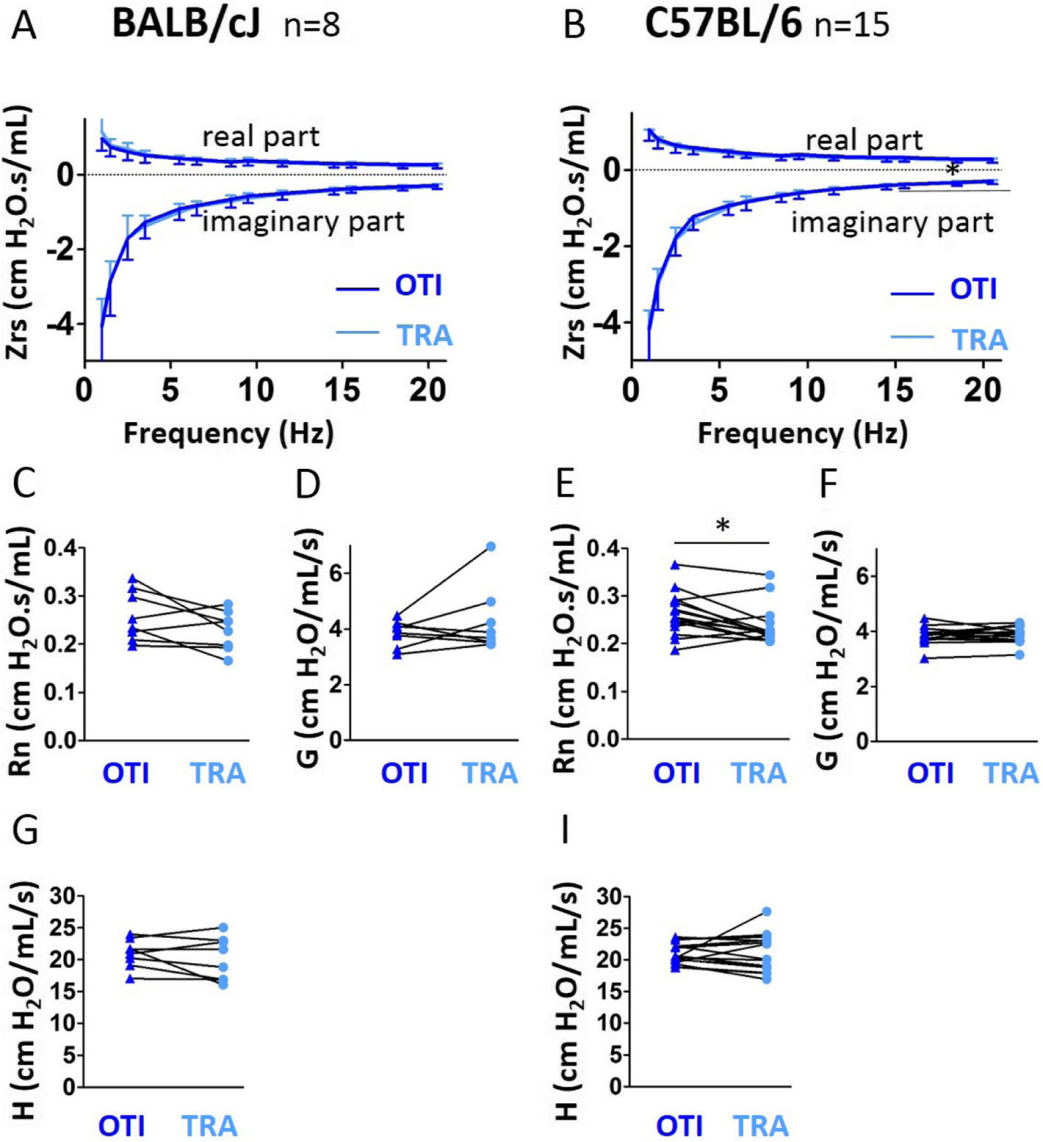
Evaluation of lung function measurement assessed by constant phase model obtained in mice using orotracheal intubation and tracheostomy, both using the intubation cannula. **a**, **b**, Average real (top) and imaginary (bottom) parts of respiratory system input impedance (Zrs) obtained in intubated (“OTI”, in blue) mice and tracheostomized (“TRA”, in red) mice. n = 8 BALB/cJ mice (**a**), n = 15 C57BL/6 J mice (**b**). Lower and upper error bars represent standard deviations respectively for intubation and tracheostomy. **c**-**i**, newtonian resistance (“Rn”, **c** and **e**), tissue damping (“G”, **d**, **f**), tissue elastance (“H”, **g**, **i**) in intubated and tracheostomized BALB/cJ mice (**c**, **d**, **g**) and C57BL/6 J mice (**e**, **f**, **i**). Data represent individual mice and are analyzed by the Wilcoxon signed-rank test or paired t tests. * *P* < 0.05

**Fig. 8:**
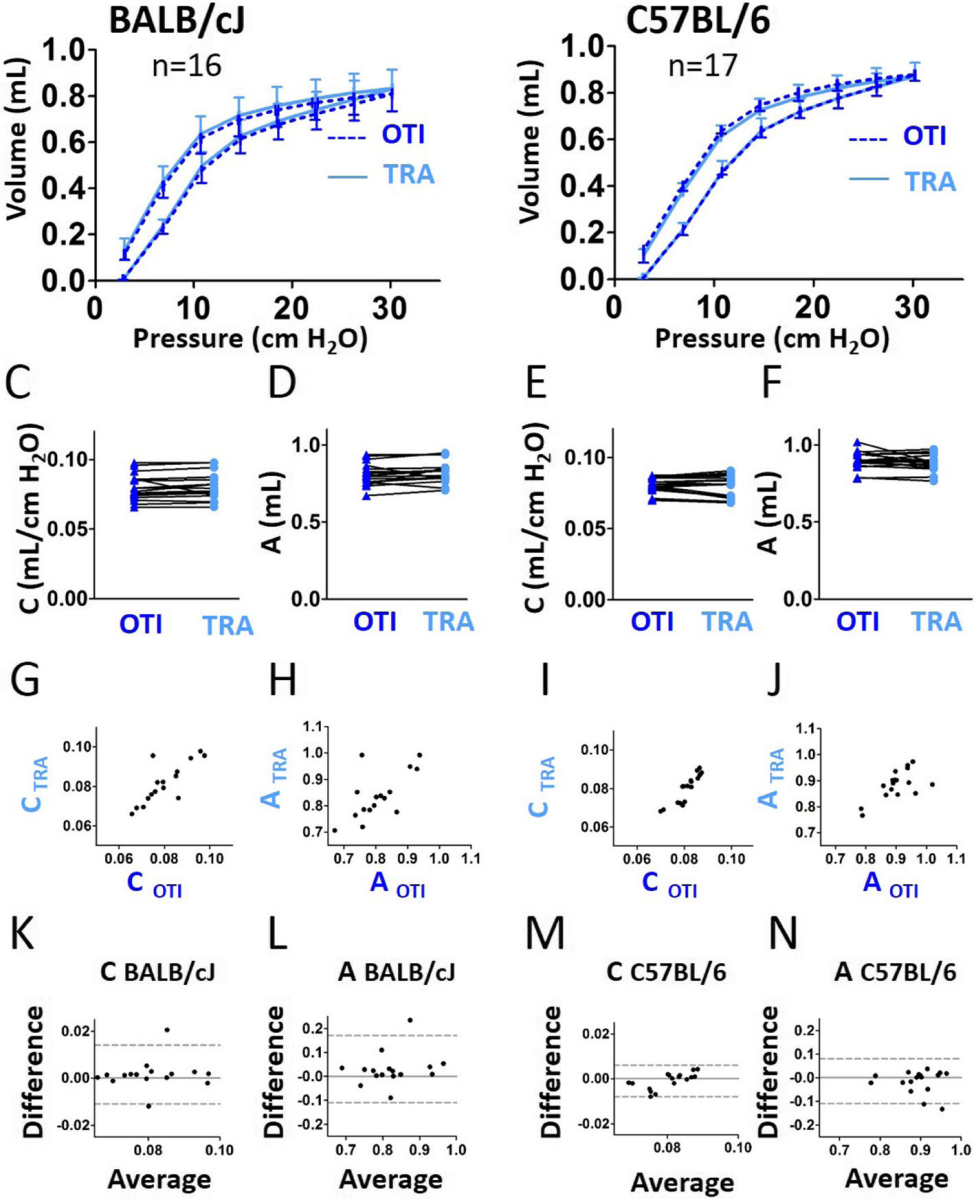
Evaluation of lung function measurement assessed by the Pressure-Volume (PV) curve maneuver in mice using orotracheal intubation and tracheostomy, both using the intubation cannula. **a**, **b**, Average PV curves of intubated (“OTI”, in blue) mice and tracheostomized (“TRA”, in red) mice. n = 16 BALB/cJ mice (**a**), n = 17 C57BL/6 J mice (**b**). Lower and upper error bars represent standard deviations for intubation and tracheostomy, respectively. **c**-**f**, compliance (“C”, **c** and **e**), estimate of inspiratory capacity (“A”, **d** and **f**) in intubated and tracheostomized BALB/cJ mice (**c**, **e**) and C57BL/6 J mice (**d**, **f**). Data represent individual mice and are analyzed by the Wilcoxon signed-rank test or paired t tests. **g**, **i**, Relationships between C measured in intubated and tracheostomized BALB/cJ (**g**) and C57BL/6 J (**i**) mice. **h**, **j**, Relationships between A measured in intubated and tracheostomized BALB/cJ (**h**) and C57BL/6 J (**j**) mice. **k**-**n**, Bland-Altman plots to compare two measurements techniques for C in BALB/cJ (**k**) and C57BL/6 J (**m**), A in BALB/cJ (**l**) and C57BL/6 J (**n**). The upper and lower limits of agreement (95% confidence interval) are shown by a gray dotted line

In intubated C57BL/6 J mice, but not in BALB/cJ mice, Rn derived from the impedance curves was significantly increased compared to tracheostomized C57BL/6 J mice (Fig. 7c, e). Our evaluation of the agreement between the two different methods by means of intra-class correlation and Bland-Altman analysis for Rn, G and H did not allow to validate the measurements of those parameters using orotracheal intubation (Fig. 7 and Additional file 1: Tables S4, S5 and S6). The PV curves obtained in intubated BALB/cJ and C57BL/6 J mice appeared almost similar to those obtained with tracheostomy (Fig. 8a-b). C and A measured in both strains (Fig. 8c-f), K and the area enclosed by the pressure volume loop measured in BALB/cJ mice (Additional file 1: Figure S7A-B) were not significantly altered in intubated compared to tracheotomized mice. The result of the combination of correlation and Bland-Altman analysis thus indicated that C, for BALB/cJ mice, and A, for both strains, could be accurately measured in intubated mice (Fig. 8g-n, and Additional file 1: Tables S1, S2 and S3).

Altogether, with the exception of Crs and C for C57BL/6 J mice, the second set of experiments allowed us to identify exactly the same subsets of parameters that are accurately evaluated in intubated BALB/cJ animals: forced vital capacity (FVC), forced expiratory volume in the first 0.1 s (FEV0.1), compliance (Crs) of the respiratory system, compliance (C) measured using PV loop, and an estimate of inspiratory capacity (A).

**Fig. 9:**
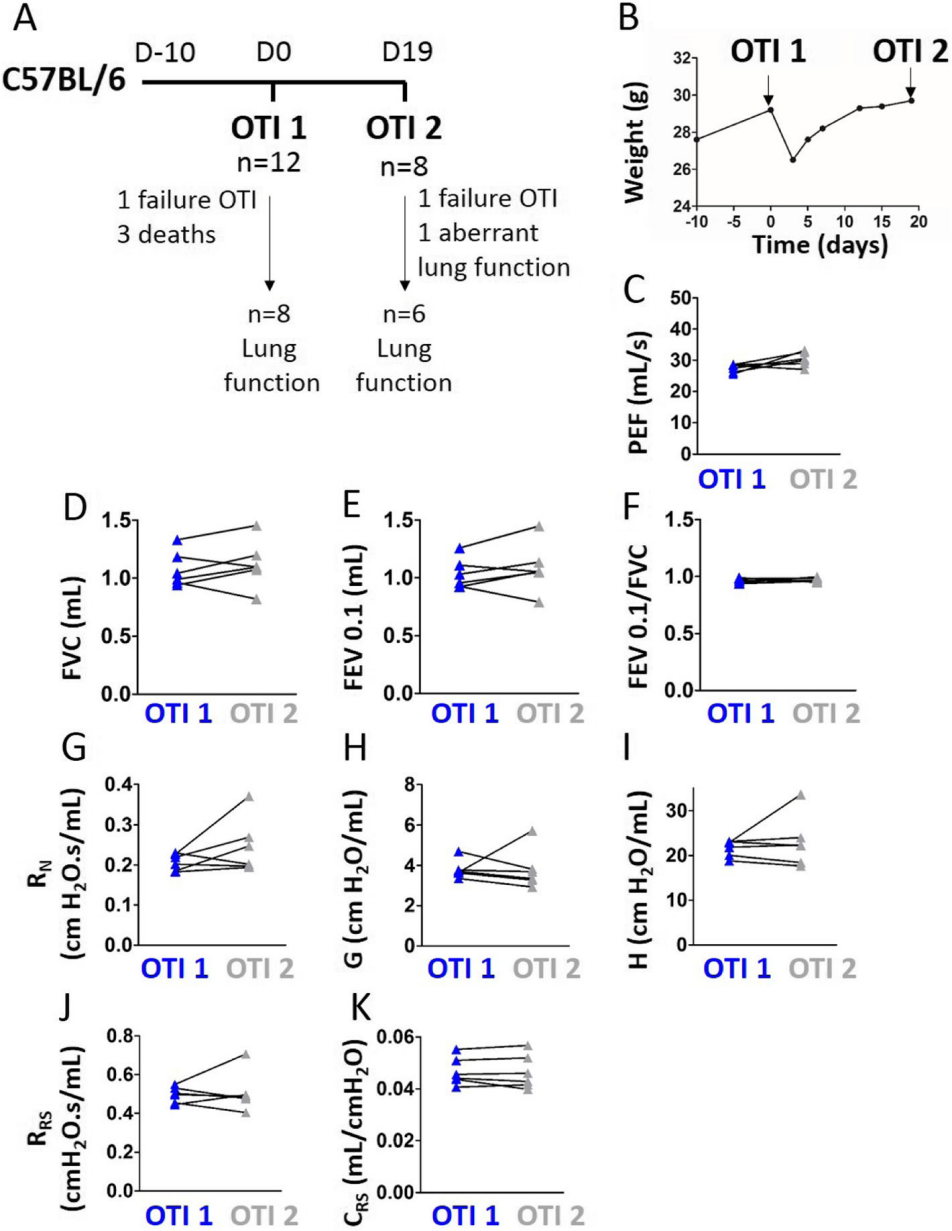
Repeated lung function measurement in intubated C57BL/6 J mice. **a**, Study protocol. Lung function measurements were performed at day 0 and 19. n represents the number of surviving mice at a given time point. **b**, Average mice weight. **c**-**k**, Peak expiratory flow (“PEF”, **c**), Forced vital capacity (“FVC”, **d**), forced expired volume over 0.1 s (“FEV0.1”, **e**), FEV0.1/FVC ratio (**f**), newtonian resistance (“Rn”, **g**), tissue damping (“G”, **h**), tissue elastance (“H”, **i**), resistance (“Rrs”, **j**) and compliance (“Crs”, **k**) of the respiratory system in intubated mice at day 0 (“OTI 1”) and day 19 (“OTI 2”). Data represent individual mice and are analyzed by the Wilcoxon signed-rank test

To test the possibility of repeated intubations, we performed sequential lung function measurements using intubation in C57BL/6 J mice with 19 days interval (Fig. 9a). Three of the 12 mice died during one of the procedures (survival rate of 75%), 2 mice (17%) could not be successfully intubated, and one mice (8%) was successfully intubated, but showed aberrant lung function data. The first orotracheal intubation is followed by weight loss (Fig. 9b). As a result, we tried without success to re-intubate mice days following the first intubation, but a second intubation was impossible to perform until the 5th day. Mice regain a normal weight around 12 days after the first intubation. In total, we could successfully measure lung function at day 0 and day 19 in 6 of the 12 mice (50%). Our results do not show any significant differences between the measurements at day 0 and 19 (Fig. 9c-k). Although no significant correlation could be evidenced for FEV0.1, the FEV0.1/FVC ratio, A, Area, Rn, G, H and Rrs measured by intubation at day 0 and day 19, probably due to the low number of mice, PEF, FVC, Crs and K measured by the first and second intubation were positively correlated (Additional file 1: Table S7). Altogether, this suggests that repetitive lung function measurements using intubation are feasible and seem to be reproducible.

## Discussion

Characterizing lung function in mice models of respiratory diseases is essential to follow variations over time. However, it has remained a real challenge to use methods that are compatible with longitudinal measurements. In particular, precise lung function evaluation in mice often requires tracheostomy, allowing only end-point measurements. Previous studies showed that lung function monitoring using intubation was feasible and could be repeated [16–20], but a thorough evaluation of the measurements obtained with intubation in comparison with those obtained by tracheostomy was lacking. Here, we have taken advantage of previously described methods of orotracheal intubation [15, 16, 19] and have compared the measurements obtained with this technique with those performed following tracheostromy. Except for the COD for the single compartment model, the CODcp and CODsk are not different between the two techniques, suggesting that the quality of the constant phase model and Salazar-Knowles model fits was not altered in intubated mice. We identified parameters that are accurately evaluated in intubated animals (i.e., FVC, FEV0.1, Crs, C and A in BALB/cJ and FVC, FEV0.1, and A in C57BL/6 J).

In contrast, we have evidenced discrepancies between intubated and tracheostomized mice, in particular during forced expiratory maneuver which deserve some methodological discussion. First, a single anesthetic protocol was used to perform both lung function measurements, using orotracheal intubation, and subsequently using tracheostomy. It is therefore possible that waning anesthetic level leading to increased tonic muscle tone of the chest wall could influence the measurements obtained during tracheostomy. However, no sign of respiratory drive was observed during the measurements, and the mice were still deeply anesthetized at the end of complete sequences of lung function measurements which tends to rule out this hypothesis to explain the discrepancies between intubation and tracheostomy. Second, unlike in the tracheostomy protocol, there was no suture sealing the wall of the trachea around the intubation cannula, leading to possible leakage problems and subsequent erroneous measurements of respiratory function. However, leakage would cause an increase in inspiratory capacity and this parameter was not different between intubated and tracheostomized mice for both strains. Third, measurements at the onset of the forced expiratory maneuver were probably biased by the intubation catheter, which had a resistance slightly higher than that of the cannula used for tracheostomy. Taking into account the cannula resistance value, we evaluated the reduction in flow rate due to increased cannula resistance, which could explain only 10% of the 40% of reduction in peak expiratory flow.

Subsequent experiments performed with the same cannula for intubation and tracheostomy showed that some of the discrepancies between intubation and tracheostomy observed previously were in fact due to the different cannula used for intubation and tracheostomy. Although the expiratory flow-volume, impedance and PV curves looked very similar in intubated and tracheostomized mice, subtle differences still persisted. In total, our evaluation of the agreement between the two different methods by means of intra-class correlation and Bland-Altman analyses allowed us to validate exactly the same subset of parameters previously identified (FVC, FEV0.1, Crs, C and A), that were accurately evaluated in intubated BALB/cJ animals. The measurements of Crs and C in intubated C57BL/6 J mice should be used carefully in this specific strain. Indeed, their use was validated by our first comparison study using different cannulas, but not the second one with the same catheters. However, the existence of a proportional bias does not prevent the use of intubation to measure C before and after an experimental challenge, on the condition that only relative changes are mentioned.

Some apparent differences between both strains can be observed. In particular, the expiratory flow-volume curve measured in intubated BALB/cJ mice was altered compared to that obtained in tracheostomized mice, while both curves were very similar in C57BL/6 J mice. Regarding Rn, as expected, taking into account that it evaluates “proximal” resistance, Rn was smaller in tracheostomized BALB/cJ whereas G and H were similar in intubated and tracheostomized BALB/cJ. A similar result was not found in C57BL/6 J since Rn was not different in intubated and tracheostomized mice. This may be a consequence of a scattering of the data in a smaller C57BL/6 J group than in the BALB/cJ group. Moreover, calculation of the threshold frequency that discriminates the central compartment alone (at high frequency) from the combination central and distal compartments (at low frequency) showed that it was different in both strains giving some support to JH Bates et al. conclusion [22] stating that there is a need for comparative studies on the oscillatory mechanics of different strains involved in mouse models of lung disease. Finally, additional experiments showed that (i) lung function measured in tracheostomized C57BL/6 J mice was, in fact, affected by previous intubation (Additional file 1: Figure S2F), (ii) variations in lung function measurements between intubation and tracheostomy were similar in both strains of mice, (iii) Rn was smaller in tracheostomized C57BL/6 J mice in a second set of experiments using the intubation catheter for tracheostomy (Fig. 7e). Of note, in this set of experiments, Rn was not significantly decreased in tracheostomized BALB/cJ mice (Fig. 7c).

We have also investigated the possibility of repeated lung function measurements. As already shown by De Vleeschauwer et al, all mice lost weight after the first intubation [20]. The large intubation cannula may induce local tracheal edema, transiently reducing the possibility of food intake. As a result, we strongly advice to keep an interval of at least one week between two lung function measurements. Although the overall survival was affected by these repeated invasive monitoring, which is limiting in a chronic animal model, our data demonstrated that repeated measurements are feasible, and seem to be reproducible.

The technique of intubation had side-effects with an increase in mortality associated to the intubation procedure. Different methods of orotracheal intubation have been proposed [9–15, 23–31]. Although all these methods seem to be successfully used in the literature, they require some expertise and considerable training with a relatively long learning curve, as already shown in human [32]. In addition, the real challenge is not mouse intubation *per se*, but intubation with a cannula compatible with lung function measurement, which must offer the best compromise between a resistance close to that of the tracheostomy cannula, sufficient flexibility to enable orotracheal intubation and a diameter close to that of the trachea to avoid air leakage. Nevertheless, it can be anticipated that mortality should be lowered as the experience of the operator increases.

## Conclusion

In conclusion, we have identified a subset lung function parameters that could accurately be evaluated in intubated mice (i.e., FVC, FEV0.1, Crs, C and A in BALB/cJ and FVC, FEV0.1, and A in C57BL/6 J). Repetitive lung function measurements by orotracheal intubation are feasible and seem to be reproducible in C57BL/6 J mice. This suggests that lung function can be reliably assessed using orotracheal intubation in mice and therefore may be used for longitudinal studies in model of chronic respiratory diseases. The ability of lung function measurement using orotracheal intubation to detect functional and pathological changes in mice remains to be tested in various pathological models. However, we strongly believe that repeated lung function measurement in intubated mice would have several advantages in longitudinal studies with limited numbers of expensive animals.

## Supplementary information

**Additional file 1: Table S1.** Association between parameters measured using tracheostomy and orotracheal intubation. **Table S2.** Agreement between the measurements of parameters by tracheostomy and orotracheal intubation. **Table S3.** Association between the difference and the average of the two methods for the measurements using tracheostomy and orotracheal intubation. **Table S4.** Association between parameters measured using tracheostomy and orotracheal intubation, both using the intubation cannula. **Table S5.** Agreement between the measurements of parameters by tracheostomy and orotracheal intubation, both using the intubation cannula. **Table S6.** Association between the difference and the average of the two methods for the measurements using tracheostomy and orotracheal intubation, both using the intubation cannula. **Table S7.** Association between parameters measured using orotracheal intubation at day 0 et day 19. **Figure S1.** Evaluation of peak expiratory flow and the FEV0.1/FVC ratio measurements obtained in mice using orotracheal intubation and tracheostomy. **Figure S2.** Evaluation of lung function measurement in C57BL/6 J mice that have been either intubated or tracheostomized. **Figure S.** Evaluation of quality of the single compartment, contant phase and Salazar-Knowles models fit. **Figure S4.** Evaluation of the curvature of the upper portion of the deflation limb of the Pressure-Volume loop and the area enclosed by the same curve, obtained in mice using orotracheal intubation and tracheostomy. **Figure S5.** Relationships between the respiratory system compliance (Crs) and the compliance (C) measured in intubated and tracheostomized BALB/cJ (A) and C57BL/6 J mice (B). **Figure S6.** Evaluation of peak expiratory flow and the FEV0.1/FVC ratio measurements obtained in mice using orotracheal intubation and tracheostomy, both using the intubation cannula. **Figure S7.** Evaluation of the curvature of the upper portion of the deflation limb of the Pressure-Volume loop and the area enclosed by the same curve, obtained in mice using orotracheal intubation and tracheostomy, both using the intubation cannula. (DOCX 757 kb)

## Data Availability

The dataset supporting the conclusions of this article is included within the article, lung function analyses are available from the corresponding author on request.

## Abbreviations

A: estimate of inspiratory capacity
C: compliance measured using PV loop
COD: Coefficients of determination for the single compartment model fit
CODcp: Coefficient of determination for the constant phase model fit
CODsk: Coefficient of determination for the Salazar-Knowles model fit
Crs: Compliance of the respiratory system
FEV0.1: Forced expiratory volume in the first 0.1 s
FOT: Forced oscillation technique
FVC: Forced vital capacity
G: tissue damping
H: tissue elastance
K: curvature of the upper portion of the deflation limb of the PV curve
NPFE: Negative pressure-driven forced expiratory
PEF: Peak expiratory flow
PV: Pressure-volume
Rn: Resistance of the central airways
Rrs: Resistance of the respiratory system
