# Supplementary material for: Determination of reliable lung function parameters in intubated mice
Source: Respir Res. 2019 Sep 14;20:211. doi: 10.1186/s12931-019-1177-9 (PMC6744631; doi:10.1186/s12931-019-1177-9)
Supplement: Supplementary file 1 — Additional file 1: Table S1. Association between parameters measured using tracheostomy and orotracheal intubation. Table S2. Agreement between the measurements of parameters by tracheostomy and orotracheal intubation. Table S3. Association between the difference and the average of the two methods for the measurements using tracheostomy and orotracheal intubation. Table S4. Association between parameters measured using tracheostomy and orotracheal intubation, both using the intubation cannula. Table S5. Agreement between the measurements of parameters by tracheostomy and orotracheal intubation, both using the intubation cannula. Table S6. Association between the difference and the average of the two methods for the measurements using tracheostomy and orotracheal intubation, both using the intubation cannula. Table S7. Association between parameters measured using orotracheal intubation at day 0 et day 19. Figure S1. Evaluation of peak expiratory flow and the FEV0.1/FVC ratio measurements obtained in mice using orotracheal intubation and tracheostomy. Figure S2. Evaluation of lung function measurement in C57BL/6 J mice that have been either intubated or tracheostomized. Figure S. Evaluation of quality of the single compartment, contant phase and Salazar-Knowles models fit. Figure S4. Evaluation of the curvature of the upper portion of the deflation limb of the Pressure-Volume loop and the area enclosed by the same curve, obtained in mice using orotracheal intubation and tracheostomy. Figure S5. Relationships between the respiratory system compliance (Crs) and the compliance (C) measured in intubated and tracheostomized BALB/cJ (A) and C57BL/6 J mice (B). Figure S6. Evaluation of peak expiratory flow and the FEV0.1/FVC ratio measurements obtained in mice using orotracheal intubation and tracheostomy, both using the intubation cannula. Figure S7. Evaluation of the curvature of the upper portion of the deflation limb of the Pressure-Volume loop and the area enclosed by [file 12931_2019_1177_MOESM1_ESM.docx]

**Determination of reliable lung function parameters in intubated mice**

Eline Bonnardel^1,2^, Renaud Prevel^1,2^, Marilyne Campagnac^1,2^, Marielle Dubreuil^1,2^, Roger Marthan^1,2,3^, Patrick Berger^1,2,3,*^, Isabelle Dupin^1,2,*^

**Online Data Supplement**

**Supplemental tables**

**Table S1 Association between mechanical parameters measured using tracheostomy and orotracheal intubation**

|  |  | **BALB/cJ mice** | | | **C57BL/6J mice** | | |
| --- | --- | --- | --- | --- | --- | --- | --- |
|  |  | | Pearson r | P value | | Pearson r | P value |
| **NPFE**  FVC (mL) | | | 0.87 | **0.0001** | | 0.68 | **0.03** |
| FEV0.1 (mL)  FEV0.1/FVC  PEF (mL/s)  **PV curve**  C (mL/cm H_2_O)  A (mL)  K(/cm H_2_O)  Area (cm H_2_O/mL)    **Single compartment model**  Crs (mL/cm H_2_O)  Rrs (cmH_2_O.s/mL)  **Constant phase model**  G (cm H_2_O/mL/s)  H (cm H_2_O/mL/s)  Rn (cmH_2_O.s/mL) | | | 0.80  0.54  -0.20  0.78  0.73  0.44  0.06  0.70  0.43  0.37  0.41  0.51 | **0.0009**  0.06  0.51  **0.0009**  **0.003**  0.11  0.83  **0.005**  0.12  0.23  0.18  0.09 | | 0.57  0.28  -0.06  0.94  0.92  0.91  0.29  0.87  -0.53  0.34  0.88  -0.28 | 0.09  0.43  0.88  **<0.0001**  **0.0002**  **0.0002**  0.41  **0.0009**  0.12  0.37  **0.001**  0.46 |
|  | |  | |  |  | |  |

NPFE, negative pressure-driven forced expiratory maneuver; PV curve, pressure-volume curve; FVC, forced vital capacity; FEV0.1, forced expired volume over 0.1 second; PEF, peak expiratory flow; C, compliance measured using PV loop, A, estimate of inspiratory capacity; K, curvature of the upper portion of the deflation limb of the PV curve; Area, the area enclosed by the PV loop; Crs, respiratory system compliance; Rrs, respiratory system resistance; G, tissue damping; H, tissue elastance, Rn, newtonian resistance. Correlation coefficient (r) and significance level (P value) were obtained by using Pearson analysis.

**Table S2 Agreement between the measurements of mechanical parameters by tracheostomy and orotracheal intubation**

|  |  | **BALB/cJ mice** | | | |  | **C57BL/6J mice** | | | |  | |
| --- | --- | --- | --- | --- | --- | --- | --- | --- | --- | --- | --- | --- |
|  |  | | Bias | SD of bias | 95% limits of agreement From… to | | | Bias | SD of bias | 95% limits of agreement From… to | |  |
| **NPFE**  FVC (mL) | | | 0.03 | 0.06 | -0.10 to 0.15 | | | 0.01 | 0.12 | -0.23 to 0.25 | |  |
| FEV0.1 (mL)  **PV curve**  C (mL/cm H_2_O)  A (mL)    **Single compartment model**  Crs (mL/cm H_2_O) | | | 0.05  -0.001  -0.001  0.001 | 0.08  0.003  0.03  0.002 | -0.10 to 0.20  -0.007 to 0.005  -0.061 to 0.063  -0.004 to 0.006 | | | 0.01  0.005  0.003  0.001 | 0.13  0.002  0.02  0.002 | -0.23 to 0.26  -0.004 to 0.005  -0.037 to 0.043  -0.002 to 0.005 | |  |

NPFE, negative pressure-driven forced expiratory maneuver; PV curve, pressure-volume curve; FVC, forced vital capacity; FEV0.1, forced expired volume over 0.1 second; C, compliance measured using PV loop, A, estimate of inspiratory capacity; Crs, respiratory system compliance; Bias, standard deviation (SD) of bias and 95% limits of agreement were obtained by using Bland-Altman analysis. Correlation coefficient (r) and significance level (P value) were obtained by using Spearman analysis.

**Table S3 Association between the difference and the average of the two methods for the measurements of mechanical parameters measured using tracheostomy and orotracheal intubation**

|  |  | **BALB/cJ mice** | | **C57BL/6J mice** | |
| --- | --- | --- | --- | --- | --- |
|  |  | Spearman r | P value | Spearman r | P value |
| **NPFE**  FVC (mL) | | 0.08 | 0.80 | -0.05 | 0.88 |
| FEV0.1 (mL)  **PV curve**  C (mL/cm H_2_O)  A (mL)    **Single compartment model**  Crs (mL/cm H_2_O) | | -0.02  0.08  0.04  0.15 | 0.86  0.79  0.89  0.61 | 0.01  0.05  0.21  0.42 | 0.99  0.88  0.56  0.23 |
|  | |  |  |  |  |

NPFE, negative pressure-driven forced expiratory maneuver; PV curve, pressure-volume curve; FVC, forced vital capacity; FEV0.1, forced expired volume over 0.1 second; C, compliance measured using PV loop, A, estimate of inspiratory capacity; Crs, respiratory system compliance; Ers, respiratory system elastance; Rrs, respiratory system resistance. Correlation coefficient (r) and significance level (P value) were obtained by using Spearman analysis.

**Table S4 Association between mechanical parameters measured using tracheostomy and orotracheal intubation, both using** **the intubation cannula**

|  |  | **BALB/cJ mice** | | | **C57BL/6J mice** | | |
| --- | --- | --- | --- | --- | --- | --- | --- |
|  |  | | Pearson r | P value | | Pearson r | P value |
| **NPFE**  FVC (mL) | | | 0.77 | **0.0005** | | 0.79 | **0.0003** |
| FEV0.1 (mL)  FEV0.1/FVC  PEF (mL/s)  **PV curve**  C (mL/cm H_2_O)  A (mL)  K(/cm H_2_O)  Area (cm H_2_O/mL)    **Single compartment model**  Crs (mL/cm H_2_O)  Rrs (cmH_2_O.s/mL)  **Constant phase model**  G (cm H_2_O/mL/s)  H (cm H_2_O/mL/s)  Rn (cmH_2_O.s/mL) | | | 0.75  0.40  0.52  0.80  0.65  0.28  0.21  0.89  0.46  0.55  0.71  0.45 | **0.0009**  0.13  0.07  **0.0002**  **0.003**  0.29  0.42  **<0.0001**  0.07  0.17  **0.047**  0.27 | | 0.80  0.22  0.35  0.90  0.65  0.36  0.07  0.91  0.29  0.67  0.58  0.64 | **0.0002**  0.40  0.36  **<0.0001**  **0.005**  0.16  0.77  **<0.0001**  0.25  **0.007**  **0.02**  **0.01** |
|  | |  | |  |  | |  |

NPFE, negative pressure-driven forced expiratory maneuver; PV curve, pressure-volume curve; FVC, forced vital capacity; FEV0.1, forced expired volume over 0.1 second; PEF, peak expiratory flow; C, compliance measured using PV loop, A, estimate of inspiratory capacity; K, curvature of the upper portion of the deflation limb of the PV curve; Area, the area enclosed by the PV loop; Crs, respiratory system compliance; Rrs, respiratory system resistance; G, tissue damping; H, tissue elastance, Rn, newtonian resistance. Correlation coefficient (r) and significance level (P value) were obtained by using Pearson analysis.

**Table S5 Agreement between the measurements of mechanical parameters by tracheostomy and orotracheal intubation, both using** **the intubation cannula**

|  |  | **BALB/cJ mice** | | | |  | **C57BL/6J mice** | | | |  | |
| --- | --- | --- | --- | --- | --- | --- | --- | --- | --- | --- | --- | --- |
|  |  | | Bias | SD of bias | 95% limits of agreement From… to | | | Bias | SD of bias | 95% limits of agreement From… to | |  |
| **NPFE**  FVC (mL) | | | -0.007 | 0.08 | -0.17 to 0.16 | | | 0.03 | 0.09 | -0.14 to 0.25 | |  |
| FEV0.1 (mL)  **PV curve**  C (mL/cm H_2_O)  A (mL)    **Single compartment model**  Crs (mL/cm H_2_O) | | | 0.03  0.002  0.03  0.002 | 0.08  0.006  0.07  0.003 | -0.12 to 0.18  -0.011 to 0.014  -0.11 to 0.17  -0.003 to 0.007 | | | 0.03  -0.001  -0.02  0.007 | 0.09  0.004  0.05  0.002 | -0.13 to 0.20  -0.008 to 0.006  -0.11 to 0.08  -0.004 to 0.005 | |  |
| **Constant phase model**  H (cm H_2_O/mL/s) | | | -0.9 | 2.4 | -5.6 to 3.8 | | | 0.12 | 2.4 | -4.6 to 4.9 | |  |

NPFE, negative pressure-driven forced expiratory maneuver; PV curve, pressure-volume curve; FVC, forced vital capacity; FEV0.1, forced expired volume over 0.1 second; C, compliance measured using PV loop, A, estimate of inspiratory capacity; Crs, respiratory system compliance; H, tissue elastance, Bias, standard deviation (SD) of bias and 95% limits of agreement were obtained by using Bland-Altman analysis.

**Table S6 Association between the difference and the average of the two methods for the measurements of mechanical parameters measured using tracheostomy and orotracheal intubation, both using** **the intubation cannula**

|  |  | **BALB/cJ mice** | | **C57BL/6J mice** | |
| --- | --- | --- | --- | --- | --- |
|  |  | Spearman r | P value | Spearman r | P value |
| **NPFE**  FVC (mL) | | -0.03 | 0.90 | -0.01 | 0.97 |
| FEV0.1 (mL)  **PV curve**  C (mL/cm H_2_O)  A (mL)    **Single compartment model**  Crs (mL/cm H_2_O) | | 0.16  0.29  0.21  0.26 | 0.56  0.28  0.43  0.33 | -0.01  0.69  0.12  0.73 | 0.64  **0.002**  0.64  **0.0008** |
| **Constant phase model**  H (cm H_2_O/mL/s) | | 0.57 | 0.15 | 0.70 | **0.003** |
|  | |  |  |  |  |

NPFE, negative pressure-driven forced expiratory maneuver; PV curve, pressure-volume curve; FVC, forced vital capacity; FEV0.1, forced expired volume over 0.1 second; C, compliance measured using PV loop, A, estimate of inspiratory capacity; Crs, respiratory system compliance; H, tissue elastance. Correlation coefficient (r) and significance level (P value) were obtained by using Spearman analysis.

**Table S7 Association between mechanical parameters measured using orotracheal intubation at day 0 et day 19**

|  |  | **C57BL/6J mice** | | |
| --- | --- | --- | --- | --- |
|  |  | | Pearson r | P value |
| **NPFE**  FVC (mL) | | | 0.89 | **0.03** |
| FEV0.1 (mL)  FEV0.1/FVC  PEF (mL/s)  **PV curve**  C (mL/cm H_2_O)  A (mL)  K(/cm H_2_O)  Area (cm H_2_O/mL)    **Single compartment model**  Crs (mL/cm H_2_O)  Rrs (cmH_2_O.s/mL)  **Constant phase model**  G (cm H_2_O/mL/s)  H (cm H_2_O/mL/s)  Rn (cmH_2_O.s/mL) | | | 0.54  -0.43  1  0.94  0.82  1  0.83  0.94  0.20  0.66  0.71  0.54 | 0.30  0.42  **0.003**  **0.02**  0.06  **0.003**  0.06  **0.02**  0.71  0.17  0.14  0.30 |
|  | |  | |  |

NPFE, negative pressure-driven forced expiratory maneuver; PEF, peak expiratory flow; PV curve, pressure-volume curve; FVC, forced vital capacity; FEV0.1, forced expired volume over 0.1 second; C, compliance measured using PV loop, A, estimate of inspiratory capacity; K, curvature of the upper portion of the deflation limb of the PV curve; Area, the area enclosed by the PV loop; Crs, respiratory system compliance; Rrs, respiratory system resistance; G, tissue damping; H, tissue elastance, Rn, newtonian resistance. Correlation coefficient (r) and significance level (P value) were obtained by using Pearson analysis.

**Supplemental figures legends**

**Fig. S1 Evaluation of peak expiratory flow and the FEV0.1/FVC ratio measurements assessed by the NPFE maneuver obtained in mice using orotracheal intubation and tracheostomy.** A-B, Peak expiratory flow (“PEF”) (A) and the FEV0.1/FVC ratio (B) in intubated (“OTI”) and tracheostomized (“TRA”) mice. Data represent individual mice and are analyzed by the Wilcoxon signed-rank test or paired t tests. * P < 0.05, *** P < 0.001.

**Fig. S2 Evaluation of lung function measurement in C57BL/6J mice that have been either intubated or tracheostomized**. A, Average expiratory flow-volume curves of intubated (“OTI”, in blue, n=20) mice and tracheostomized (“TRA”, in red, n=20) C57BL/6J mice. Lower and upper error bars represent standard deviations respectively for intubation and tracheostomy. B-J, Peak expiratory flow (“PEF”, B), Forced vital capacity (“FVC”, C), forced expired volume over 0.1 second (“FEV0.1”, D), FEV0.1/FVC ratio (E), newtonian resistance (“Rn”, F), tissue damping (“G”, G), tissue elastance (“H”, H), resistance (“Rrs”, I) and compliance (“Crs”, J) of the respiratory system in intubated (“OTI”) and tracheostomized (“TRA”) mice. Data represent individual mice and are analyzed by Mann–Whitney or unpaired t tests. ** P < 0.01, *** P < 0.001.

**Fig. S3 Evaluation of quality of the single compartment, contant phase and Salazar-Knowles models fit.** A-C, Comparaison of the coefficients of determination for the single compartment model fit (COD, A), coefficient of determination for the constant phase model fit (CODcp, B), coefficient of determination for the Salazar-Knowles model (CODsk, C), in intubated (“OTI”) and tracheostomized (“TRA”) mice. Data represent individual mice and are analyzed by the Wilcoxon signed-rank test or paired t tests. * P < 0.05, *** P < 0.001.

**Fig. S4 Evaluation of the curvature of the upper portion of the deflation limb of the Pressure-Volume loop and the area enclosed by the same curve, obtained in mice using orotracheal intubation and tracheostomy.** A-D, Comparaison of the variables curvature of the upper portion of the deflation limb of the Pressure-Volume loop (“K”, A), and the area enclosed by the Pressure-Volume loop (“Area”, B) in intubated (“OTI”) and tracheostomized (“TRA”) mice. n=14 BALB/cJ mice, n=10 C57BL/6J mice. Data represent individual mice.

**Fig. S5 Relationships between the respiratory system compliance (Crs) and the compliance (C) measured in intubated and tracheostomized BALB/cJ (A) and C57BL/6J mice (B).** The reference slopes (C=Crs) are shown by a gray line. Correlation coefficient (r) and significance level (P value) were obtained by using Pearson analysis.

**Fig. S6 Evaluation of peak expiratory flow and the FEV0.1/FVC ratio measurements assessed by the NPFE maneuver obtained in mice using orotracheal intubation and tracheostomy, both using** **the intubation cannula.** A-B, Peak expiratory flow (“PEF”) (A) and the FEV0.1/FVC ratio (B) in intubated (“OTI”) and tracheostomized (“TRA”) mice. n=16 BALB/cJ mice, n=16 C57BL/6J mice. Data represent individual mice and are analyzed by the Wilcoxon signed-rank test or paired t tests. * P < 0.05.

**Fig. S7 Evaluation of the curvature of the upper portion of the deflation limb of the Pressure-Volume loop and the area enclosed by the same curve, obtained in mice using orotracheal intubation and tracheostomy, both using** **the intubation cannula.** A-D, Comparaison of the variables curvature of the upper portion of the deflation limb of the Pressure-Volume loop (“K”, A), and the area enclosed by the Pressure-Volume loop (“Area”, B) in intubated (“OTI”) and tracheostomized (“TRA”) mice. n=16 BALB/cJ mice, n=17 C57BL/6J mice. Data represent individual mice and are analyzed by the Wilcoxon signed-rank test or paired t tests. ** P < 0.01.

**Fig. S1**

**
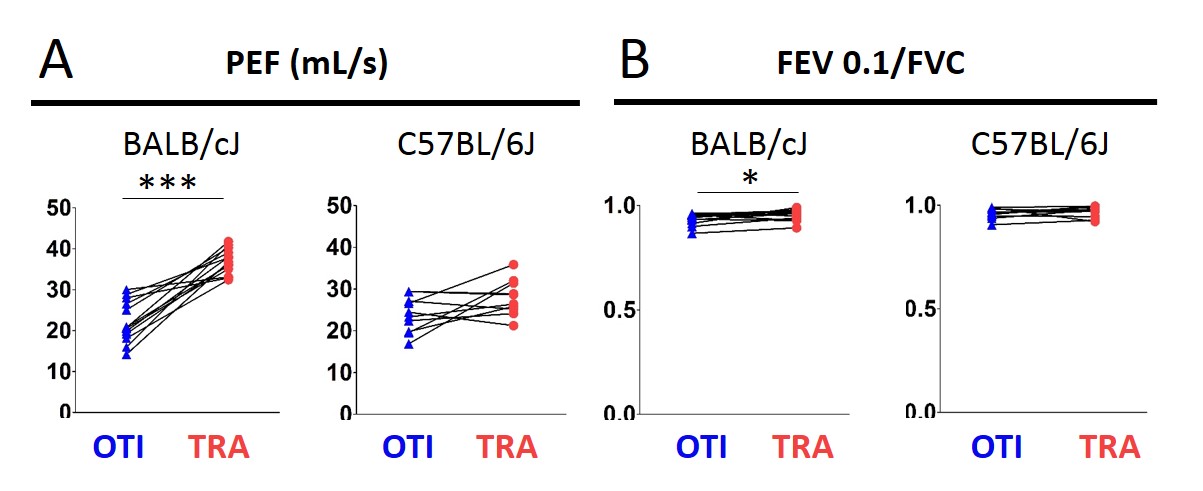
**

**Fig. S2**

**
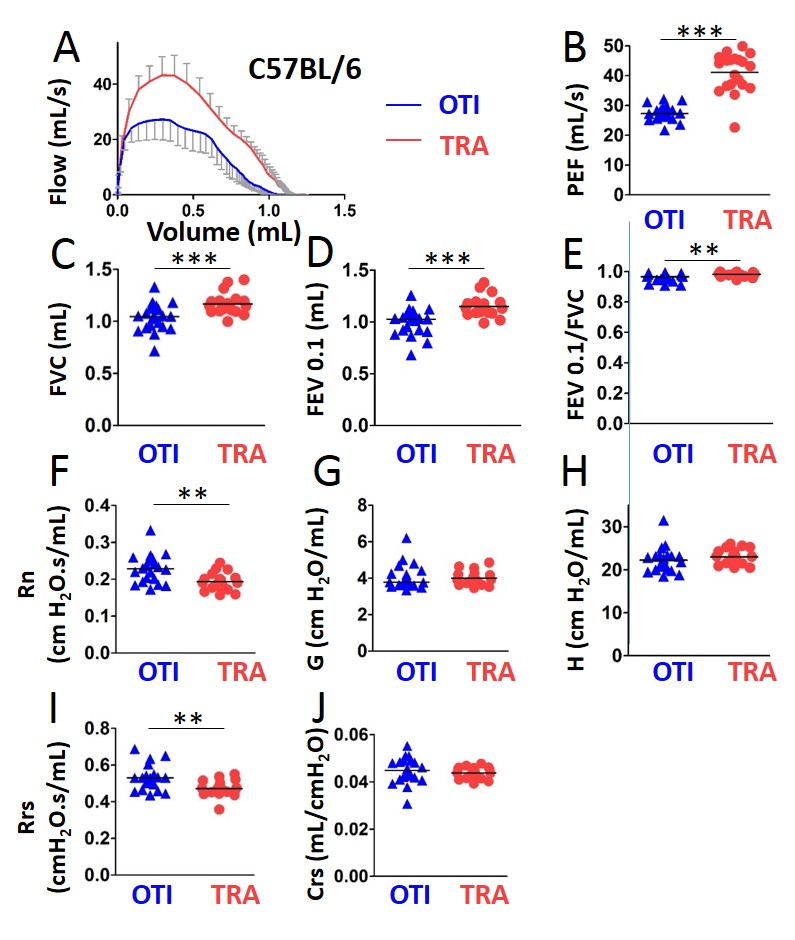
**

**Fig. S3**

**
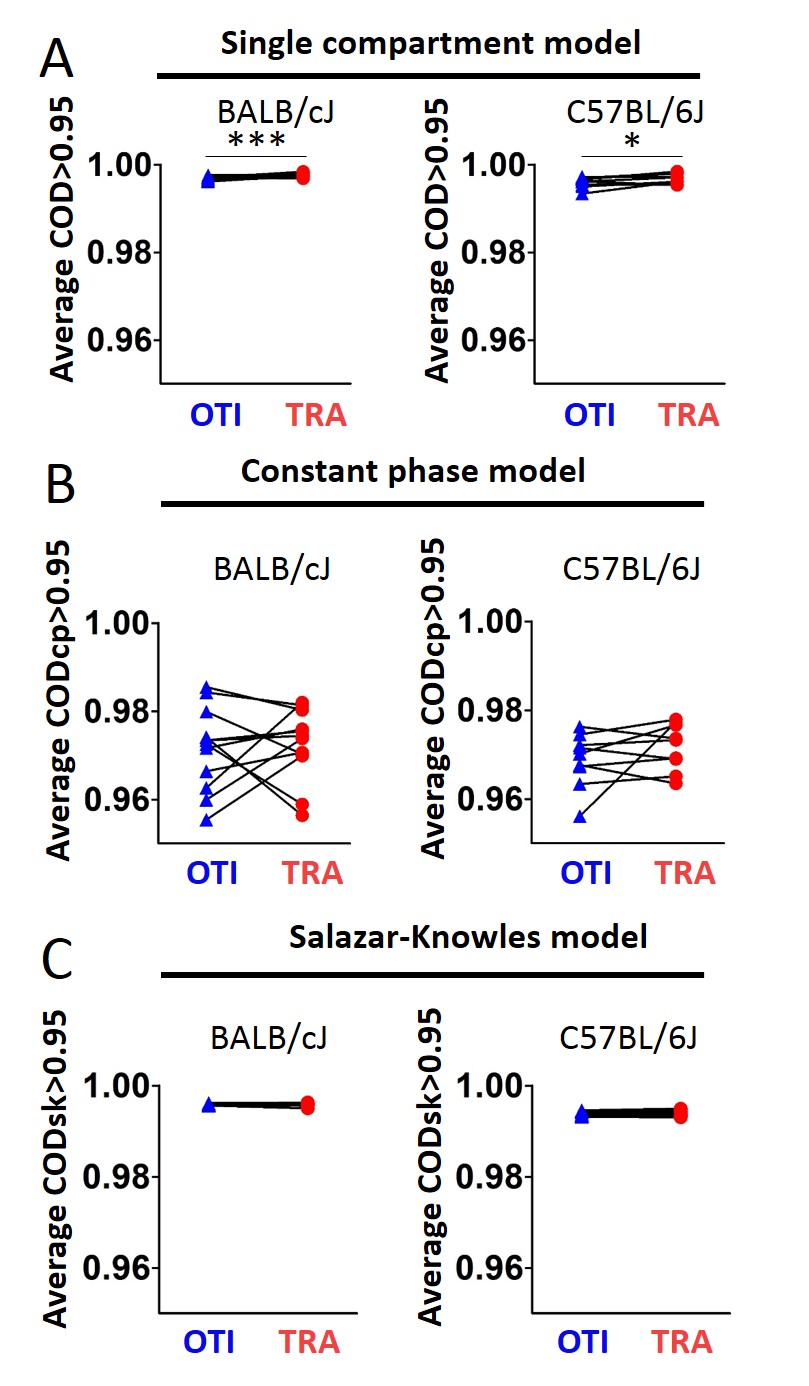
**

**Fig. S4**


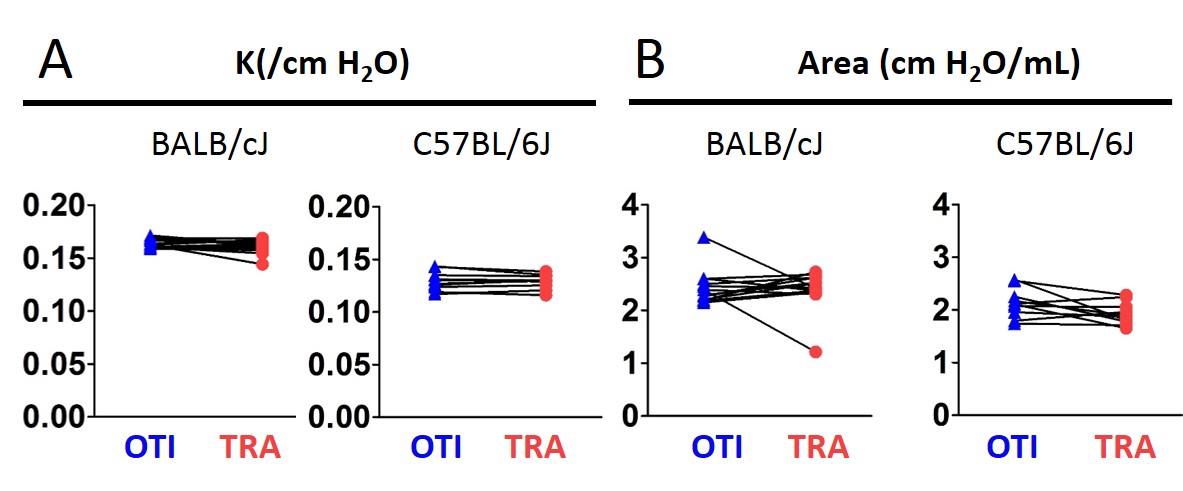


**Fig. S5**

**
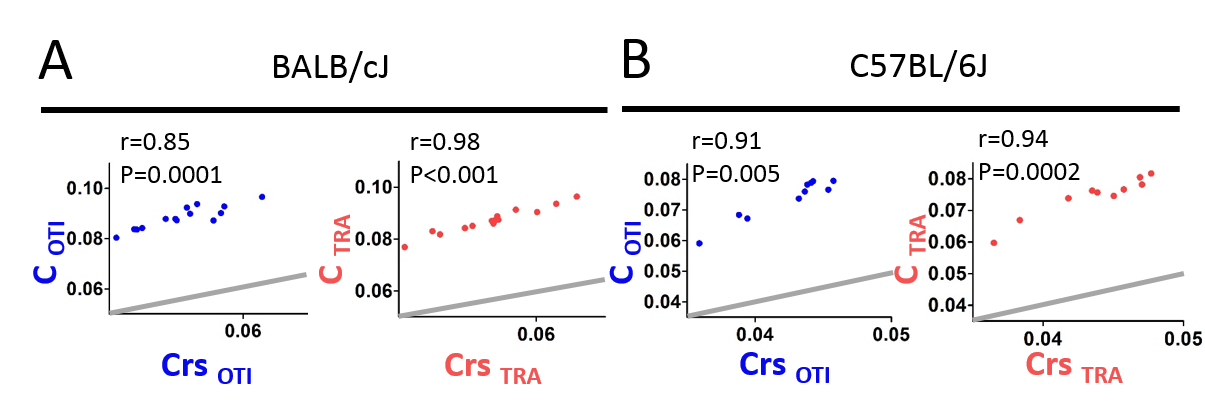
**

**Fig. S6**

**
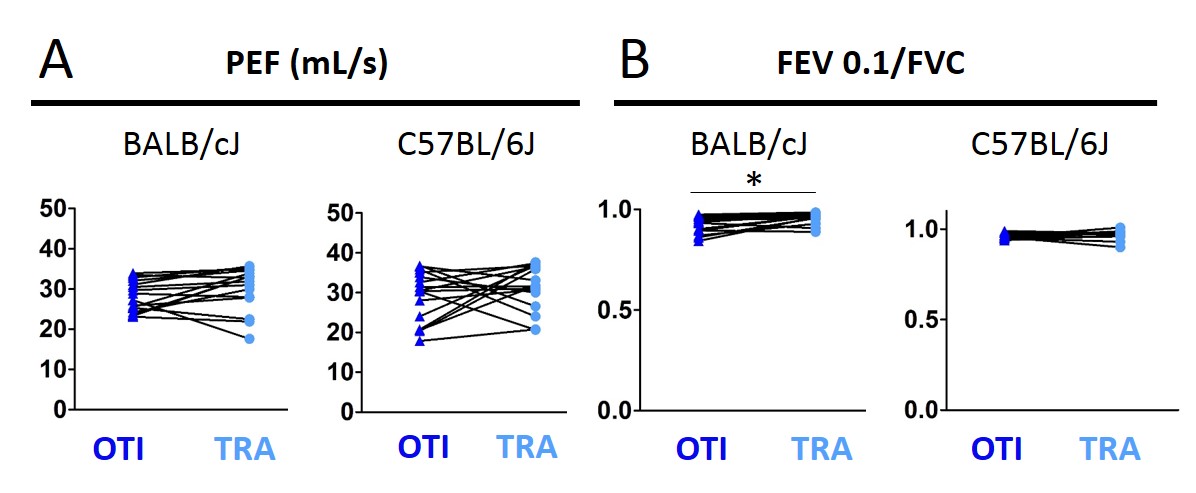
**

**Fig. S7**

**
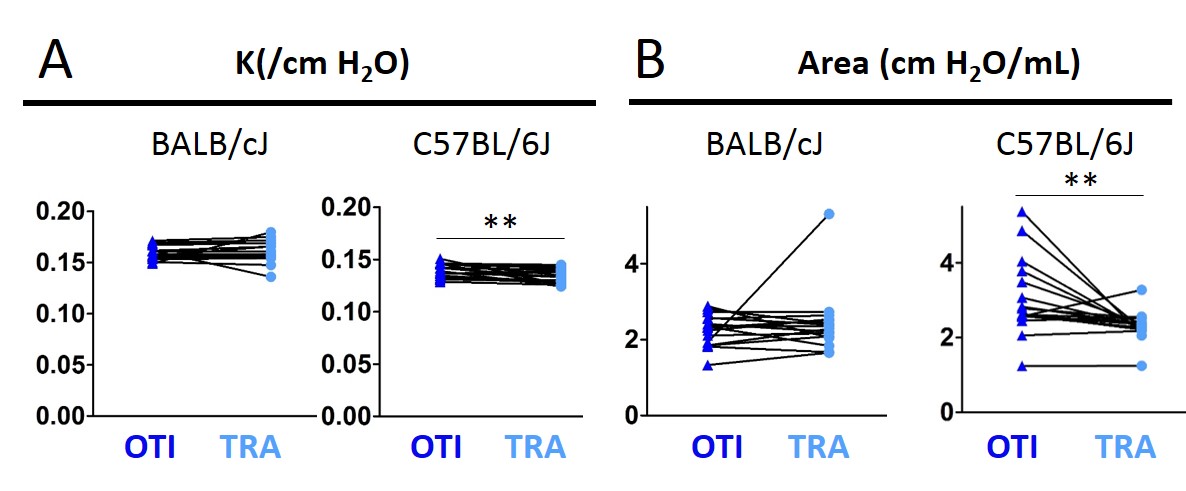
**
